# Supplementary material for: Teachers’ perceptions of the differential impacts of a universal, school-based social and emotional learning intervention: A thematic framework analysis
Source: PLoS One. 2025 Jul 22;20(7):e0328482. doi: 10.1371/journal.pone.0328482 (PMC12282926; doi:10.1371/journal.pone.0328482)
Supplement: S1 — (DOCX) [file pone.0328482.s001.docx]

**Teacher Interview Schedule (semi-structured) (20-40 minutes)**

**Interview data: aims**

1. To explore, understand and explain the processes of implementation of PATHS in English educational contexts
2. To triangulate with and support interpretation of observation and impact data

Data needed for:

1. Examining processes of implementation

- Fidelity – the extent to which the school is adhering to the intended treatment model
- Dosage - how much of session delivered; number of sessions
- Quality – how well different PATHS components are delivered
- Participant responsiveness – the degree to which children and their parents engage with the intervention
- Programme reach – rate and scope of participation
- Monitoring of control conditions
- Adaptation – the nature and extent of changes made to the intervention

1. Identification of context specific factors affecting implementation
2. Evaluating the feasibility of the future implementation of PATHS in English educational contexts

**Preamble**

1. Check that the interviewee has received the **information sheet** **and consent form** and understands the project and his/her role in it.

**Ask**: Have you any questions about the project?

1. Emphasise that:

- The research team is speaking to a range of people involved in PATHS eg senior management, teachers, pupils at all of our (23) PATHS schools
- We are interested in individual experiences and thoughts about PATHS, both positive and negative… “this is your opportunity to make your voice heard on PATHS … your comments may be helpful to others in your position at other schools at a later date”
- However, we combine all the data we collect to provide an overall picture of PATHS and its implementation and any comments in the report are attributed very generally, for example, as “A (Year 3) teacher commented that…” . Any comments/opinions will not be reported back to schools

**Ask**: Have you any questions about how we use your comments?

Ethics:

Remind interviewee:

- The interview will take about 30 minutes.
- You do not have to answer any questions that you are not comfortable with
- You can stop at any time, no explanation needed
- If any question doesn’t make sense, ask for an explanation

**Ask**: Is it alright to record the interview? The transcript will only be seen by those working on the project. I will send you a copy too if you wish.

**Ask:** Are you able/willing to sign the consent form?

**Explain procedure**:

I will begin the interview with my name, the date, time and the identifying code we have assigned to your school - this is just to keep the recordings organised. All your details will be anonymised when the data is transcribed.

The first question will be about your role in school, followed by general questions about social and emotional learning in school, then moving on to PATHS more specifically

**Ask**: Have you any questions before we start?

**Ask**: Is it OK for me to start recording now?

**Interview schedule**

State researcher’s name, date, time, school identifying code *(for data management)*

Can I just ask you to confirm your roles at school……..

……and in relation to PATHS *(eg Y3 teacher, co-ordinator, etc)*

1. **Usual practice (Implementation - programme differentiation)**

*Aims: to clarify foundations for PATHS and school ethos round SEL; perceptions of benefits of PATHS/SEL; perceptions of need for PATHS/SEL; previous practice around social-emotional learning, whether starting PATHS has been integrated or resulted in changes to this*

1. Why has the school decided to implement PATHS?

Looking for information about:

- - What sort of outcomes/change is the school aiming for? Is there a shared understanding?
  - Are there specific needs within the school that PATHS is expected to address/meet?
  - Whose decision was it to adopt PATHS?

1. What was done in school to develop social and emotional skills before you started doing PATHS?

- Do you still do this?
- Is/was this the whole school or just within your classroom?

Looking for information about:

1. Is PATHS part of a range of similar programmes/strategies? How does PATHS build on other local or national programmes/ interventions within school?
2. Has PATHS replaced previous programmes/ interventions/ approaches (eg SEAL)? Is it delivered alongside them? Are they integrated?
3. PATHS is just in KS2 – what does the rest of school do?
4. How would you describe the overall profile of PATHS in your school?

- Is it just classroom teachers in Y3-5 (Y4-6) that are involved?
- How involved is the headteacher? Senior management team?

Looking for information about:

1. Type of HT/SMT support

- verbal only?
- Active eg training time allowed, curriculum time allowed, included in planning etc

1. **Implementation (dosage, adaptation)**

*Aims: clarify implementation dosage and fidelity; modifications or adaptations and reasons for them; generalisation (link to quality)*

1. How long have you been implementing PATHS?
2. How often do you teach PATHS? *Ask for example*
3. Is this a timetabled session? Same time every week?

Looking for:

- is timetabling is a problem
- (gently probe) status of PATHS? Competing priorities?

1. Are all pupils in the class present for PATHS?

Looking for:

- Participant reach
- Is the PATHS session used as withdrawal time? If so, do these pupils have PATHS at another time?
- Do some pupils have a more targeted approach eg SEAL small group work, nurture group? Is this in addition or instead of PATHS?

1. Are you able to cover all the lesson content in the time available? How long is a lesson on average?

Ask for examples

Looking for (probe gently):

- Whether skipping content and why eg competing priorities, lack of time, low status of PATHS

1. Have you repeated any lessons?

Ask for examples; gently probe reasons

1. Have you skipped any lessons?

Ask for examples; gently probe reasons

1. Have you been able to use PATHS outside of the specific lessons/ in other subjects (teachable moments)?

Ask for examples eg which lessons, which concepts, in what ways?

1. Have you or the pupils been able to apply/generalise from PATHS in the classroom?

Ask for examples eg do pupils use control signals, fingers linked, feelings faces, compliments, golden rule

1. Have you/the pupils been able to apply/generalise from PATHS outside the classroom eg playtime?

Ask for examples eg do pupils use control signals, fingers linked, feelings faces, compliments, golden rule

1. **Attitudes to PATHS specifically**

*Aims: clarify teacher and pupil attitudes to PATHS, including perceptions of impact; clarify fidelity and dosage, pupil responsiveness; describe and/or explain modifications or adaptations; inform interpretation of process data; inform future roll-out of PATHS in UK context*

1. What do you think about the PATHS **lessons and structure**?
   - How useful do you find the lesson plans?
   - How much preparation is needed?

Ask for examples; probe for explanations eg why like/don’t like scripted lessons? Looking for (gently probe):

- - Do you follow the plans exactly? Make adaptations?
  - Is it useful to have everything prepared? What would be more useful?
  - What do you think about the order of the lessons/structure of the programme? Have you changed the order around at all?

Looking for:

- Adaptations or changes
- Ask for examples – is this proactive, intended to enhance engagement and responsiveness? Is this reactive eg due to barriers (programme resources, lack of time?)
  - How familiar are the concepts, strategies?

Looking for:

- Changes to usual practice, foundations for PATHS
  - (*If Y4/5/6*) How useful/necessary were the Jump Start lessons? *Ask for examples*

1. What do you think about the PATHS **resources** *(if not included above)*

- How appropriate/suitable are the resources?

Ask for examples eg availability of resources, age-level, particular class, SEN, emotional level, suitability for English context?

Looking for:

- How much do you adapt/make changes to the lessons?
- Ask for examples – want to identify whether this changes are proactive, intended to enhance engagement and responsiveness or reactive eg due to barriers (programme resources, time?)
- How useful have you found the parent (send-home) activities?
  - How useful has PATHS been for meeting specific needs in your class?

Ask for examples eg improving relationships, empathy, inclusion, managing emotions, understanding feelings

- - Are there any aspects of PATHS that you have found particularly useful for your class?

Ask for examples eg pupil of the day, compliments, Golden Rules, talking about feelings, control signals, fingers linked

- - Are there any aspects of PATHS that you have found not useful/appropriate?

Ask for examples eg pupil of the day, compliments, Golden Rules, talking about feelings, control signals, fingers linked

1. What do the pupils in your class think about PATHS?
   - Do they look forward to doing PATHS?
   - Are they engaged by/do they enjoy the lessons?
   - Are there any particular aspects they like?
   - Are some groups more responsive than others *(eg SEN, EBD, quiet/withdrawn)*?
   - PATHS has been designed for all the children in the class; have you found that it is useful for some groups more than others? *(eg EAL, SEN, EBD, withdrawn)*

Ask for specific examples (positive and negative)

1. **Perceptions of impact**: Has PATHS made a difference to your pupils? All pupils, or some groups of pupils particularly*?* The school more widely?

NB Acknowledge that may be too early to ask

Ask for examples eg Improved relationships, social skills, understanding of emotions, behaviour, self-control, confidence and participation (eg quiet pupils more prepared to participate), classroom climate/ethos/atmosphere, learning, motivation for learning, attendance, SEN

1. **Skills/knowledge/self-efficacy**

*Aims: teacher perceptions of self-efficacy, confidence, competence, skills and/or knowledge to implement PATHS; attitudes to training – quantity/quality, timing, content, utility etc; attitudes to support/coaching model - quantity/quality, timing, frequency, type of support available, utility etc. (NB to inform future roll-out)*

1. **Training** The PATHS programme provides a training package for teachers, with one day of training as you begin to deliver PATHS and a top-up half-day at the beginning of the second term. Were you able to attend the training?

Clarify whether first day, second (top-up) half-day or both

If yes:

- How useful did you find this? What particular aspects were useful?
- Was there anything missing?
- What additional/alternative training might have been useful?
- Were you required to ‘cascade’ the training/ brief colleagues?

Ask for examples (differentiate between first/second days)

If no:

- - Did any colleagues attend the training? Who?
  - Did they cascade the training/brief you on the training later at school?
  - How useful was this?

Ask for examples (differentiate between first/second days)

1. *(If appropriate)* Are you planning to attend the second training day? *(why/why not?)*
2. Have you had any additional training relating to PATHS specifically?
3. Have you had any other opportunities for training/professional development around social and emotional learning?

Ask for examples

1. **PP on-going support (coaching model)** In addition to the initial training, the PATHS programme includes ongoing support from a PATHS psychologist who has been assigned to your school. How useful have you found this ongoing support?
2. Would you like to see more support?
3. Would you like to see less support?
4. Would you like to see different types of support?

Ask for examples

1. How important do you feel it is to have access to ongoing support?

**E. Factors affecting implementation**

*Aims: factors influencing implementation; operation of factors as barriers or/and facilitators; responses to barriers (eg disregarded, re-active/pro-active adaptations)*

The list below outlines the key anticipated factors that may influence the implementation of PATHS at programme, classroom and school levels (there is likely to be interaction across levels). The questions above should have addressed most of these; however, please be aware of these factors so that answers may be probed or questions revisited if necessary.

Factors potentially affecting implementation

1. Programme level:

- suitability of resources

1. Teacher level:

- self-efficacy
- knowledge and skill proficiency
- level of training
- external support
- support from colleagues
- curriculum time
- preparation time
- attitude/buy-in – do not perceive need for or benefits of PATHS; not compatible with teaching style

1. Pupil level:

- Meets needs
- Engaged, responsive (appropriate resources)
- Classroom climate/pupil behaviour impede implementation

1. School level

- Prior positive/negative involvement with similar approaches and existing climate supportive/not supportive of SEL/PATHS approach
- PATHS integrated with other aspects of curriculum
- Head teacher and senior management team actively supportive of PATHS
- Head teacher and senior management state that supportive but not actively demonstrating support (*status of PATHS within school)*
- Sufficient resources allocated – classroom/curriculum time
- PATHS integrated with other aspects of school-life *posters just in classrooms or across school? Whole-staff awareness of PATHS (other than teachers directly involved in delivery, including eg lunchtime staff)*

1. How easy has it been to implement PATHS?

- Is there anything about your school that has made it easier?

Ask for specific examples (positive and negative)

1. Have there been any challenges to the implementation of PATHS?

Ask for specific examples (positive and negative)

**F.Sustainability**

*Aims: attitudes towards PATHS and change over time; sustainability*

1. The PATHS project runs for two years; how likely do you think it is that you will continue with PATHS after this?

- The entire programme?
- Particular lessons?
- PATHS framework (structure) but with amended lessons?
- Key aspects of PATHS? (*Ask for examples)*

Ask for specific examples (positive and negative)

Eg PPoD, compliments, golden rules, feelings faces

1. **Summarising experience**

*Aims: tap attitudes, beliefs, unanticipated experiences and factors*

As you know, the project is examining how well PATHS works in English schools. If it is successful, then it may be rolled out to more schools. Based on your experiences of PATHS so far, what advice would you give to a teacher in another school who has just been told she/he has to implement PATHS next term?

Ask for specific examples if appropriate (positive and negative)

1. **Closing the interview:**

*Aims: unanticipated experiences, factors etc; emergent themes*

1. Is there anything that you would like to add?
2. Is there anything that you think I should have asked you about, or missed out?

**ASK:** Do you have any questions?

Thank you very much for your help and time. I will now turn off the recorder.
