# Supplementary material for: Teachers’ perceptions of the differential impacts of a universal, school-based social and emotional learning intervention: A thematic framework analysis
Source: PLoS One. 2025 Jul 22;20(7):e0328482. doi: 10.1371/journal.pone.0328482 (PMC12282926; doi:10.1371/journal.pone.0328482)
Supplement: S2 — (DOCX) [file pone.0328482.s002.docx]

**Supplemental file 2**

*Child-level influences on outcomes: studies to inform theoretical framework*

| **Child-level influence** |  |  | |  |  |  |  |  | | |
| --- | --- | --- | --- | --- | --- | --- | --- | --- | --- | --- |
| **Gender** | Humphrey et al. (2022)  *The Good Behaviour Game (GBG)*  Boys – iatrogenic effects | Sullivan et al. (2017)  OBPP & Se*cond Step* (combined program) – Boys – externalising | | Coelho et al. (2015)  *Positive Attitude*  Girls - anxiety | Raimundo et al. (2013)  *Slowly but Steadily*  Boys - aggressiveness | Holen et al. (2012)  *Zippy’s Friends*  Girls - depression | Pössel et al. (2011)  *LARS& LISA*  Girls - coping | | Witvliet et al. (2009)  *GBG*  Boys – externalising |  |
| **SES** | Mischenko et al. (2022)  *Compassionate*  *Schools (CSP)* | | Vroom et al. (2020)  *Life Skills* | Wigelsworth et al. (2018)  *FRIENDS for Life* | Murray et al. (2014)  *Incredible Years* | Wigelsworth et al. (2013)  *Social and emotional aspects of learning (SEAL)* | Holen et al. (2012)  *Zippy’s Friends* | Holsen et al. (2009)  *Steg for Steg* | | |
| **Ethnicity** | Lee et al., (2023)  *TOOLBOX* | Mischenko et al. (2022)  *CSP* | | Vroom et al. (2020)  *Life Skills* |  |  |  |  | | |
| **Disability status** | Humphrey et al. (2018)  *PATHS* | Sullivan et al. (2017)  OBPP & Se*cond Step* (combined program) | |  |  |  |  |  | | |
| **Language status** | Humphrey et al. (2018)  *PATHS* |  | |  |  |  |  |  | | |
| **Baseline**  **Difficulties** | Humphrey et al. (2022)  *GBG* | Clarke et al. (2021)  Systematic  review | | Carroll et al. (2020)  *Kool Kids* | Low et al. (2016)  *Second Step* | Spilt et al., (2013)  *GBG* | Malti et al., (2011)  *PATHS* |  | | |
| **Baseline competency** | Clarke et al. (2021)  Systematic review | Novak et al. (2017)  *PATHS* | | Gibson et al. (2015)  *PATHS* | Maggin and Johnson (2014)  Meta-analysis of *FRIENDS* | Spilt et al. (2013)  *GBG* |  |  | | |
| **Home environment** | Mischenko et al. (2022)  *CSP* | Honess and Hunter, (2014)  *PATHS* | | Wigelsworth et al. (2013)  *SEAL* |  |  |  |  | | |

**References**

Carroll, A., Houghton, S., Forrest, K., McCarthy, M., & Sanders-O’Connor, E. (2020). Who benefits most? Predicting the effectiveness of a social and emotional learning intervention according to children’s emotional and behavioural difficulties. *School Psychology International*, *41*(3), 197–217. https://doi.org/10.1177/0143034319898741

Clarke, A., Sorgenfrei, M., Mulcahy, J., Davie, P., Freidrich, C., & McBride, T. (2021). *Adolescent mental health: A systematic review of the effectiveness of school-based interventions.* (p. 87). Early Intervention Foundation. https://www.eif.org.uk/report/adolescent-mental-health-a-systematic-review-on-the-effectiveness-of-school-based-interventions

Coelho, V., Sousa, V., Raimundo, R., & Figueira. (2015). The impact of a Portuguese middle school social–emotional learning program. *Health Promotion International*. https://doi.org/10.1093/heapro/dav064

Gibson, J. E., Werner, S. S., & Sweeney, A. (2015). EVALUATING AN ABBREVIATED VERSION OF THE PATHS CURRICULUM IMPLEMENTED BY SCHOOL MENTAL HEALTH CLINICIANS: Abbreviated PATHS by School Mental Health Clinicians. *Psychology in the Schools*, *52*(6), 549–561. https://doi.org/10.1002/pits.21844

Holen, S., Waaktaar, T., Lervåg, A., & Ystgaard, M. (2012). The effectiveness of a universal school-based programme on coping and mental health: A randomised, controlled study of Zippy’s Friends. *Educational Psychology*, *32*(5), 657–677. https://doi.org/10.1080/01443410.2012.686152

Holsen, I., Iversen, A. C., & Smith, B. H. (2009). Universal Social Competence Promotion Programme in School: Does it Work for Children with Low Socio-Economic Background? *Advances in School Mental Health Promotion*, *2*(2), 51–60. https://doi.org/10.1080/1754730X.2009.9715704

Honess, A., & Hunter, D. (2014). Teacher perspectives on the implementation of the PATHS curriculum. *Educational Psychology in Practice*, *30*(1), 51–62. https://doi.org/10.1080/02667363.2013.869490

Humphrey, N., Hennessey, A., Lendrum, A., Wigelsworth, M., Turner, A., Panayiotou, M., Joyce, C., Pert, K., Stephens, E., Wo, L., Squires, G., Woods, K., Harrison, M., & Calam, R. (2018). The PATHS curriculum for promoting social and emotional well-being among children aged 7–9 years: A cluster RCT. *Public Health Research*, *6*(10), 1–116. https://doi.org/10.3310/phr06100

Humphrey, N., Hennessey, A., Troncoso, P., Panayiotou, M., Black, L., Petersen, K., Wo, L., Mason, C., Ashworth, E., Frearson, K., Boehnke, J. R., Pockett, R. D., Lowin, J., Foxcroft, D., Wigelsworth, M., & Lendrum, A. (2022). The Good Behaviour Game intervention to improve behavioural and other outcomes for children aged 7–8 years: A cluster RCT. *Public Health Research*, *10*(7), 1–100. https://doi.org/10.3310/VKOF7695

Lee, J., Shapiro, V. B., & Kim, B.-K. E. (2023). Universal School-Based Social and Emotional Learning (SEL) for Diverse Student Subgroups: Implications for Enhancing Equity Through SEL. *Prevention Science*, *24*(5), 1011–1022. https://doi.org/10.1007/s11121-023-01552-y

Low, S., Smolkowski, K., & Cook, C. (2016). What Constitutes High-Quality Implementation of SEL Programs? A Latent Class Analysis of Second Step® Implementation. *Prevention Science*, *17*(8), 981–991. https://doi.org/10.1007/s11121-016-0670-3

Maggin, D. M., & Johnson, A. H. (2014). A Meta-Analytic Evaluation of the FRIENDS Program for Preventing Anxiety in Student Populations. *Education and Treatment of Children*, *37*(2), 277–306. https://doi.org/10.1353/etc.2014.0018

Malti, T., Ribeaud, D., & Eisner, M. P. (2011). The Effectiveness of Two Universal Preventive Interventions in Reducing Children’s Externalizing Behavior: A Cluster Randomized Controlled Trial. *Journal of Clinical Child & Adolescent Psychology*, *40*(5), 677–692. https://doi.org/10.1080/15374416.2011.597084

Mischenko, P. P., Nicholas‐Hoff, P., Schussler, D. L., Iwu, J., & Jennings, P. A. (2022). Implementation barriers and facilitators of a mindfulness‐based social emotional learning program and the role of relational trust: A qualitative study. *Psychology in the Schools*, *59*(8), 1643–1671. https://doi.org/10.1002/pits.22724

Murray, Rabiner, & Carrig. (2014). *Grade level effects of the incredible years teacher training program on emotion regulation and attention.* Society for Research on Educational Effectiveness. https://manchester.idm.oclc.org/login?url=https://www.proquest.com/reports/grade-level-effects-incredible-years-teacher/docview/1773217614/se-2

Novak, M., Mihić, J., Bašić, J., & Nix, R. L. (2017). PATHS in Croatia: A school-based randomised-controlled trial of a social and emotional learning curriculum: PATHS in Croatia: A school-based randomised-controlled trial. *International Journal of Psychology*, *52*(2), 87–95. https://doi.org/10.1002/ijop.12262

Pössel, P., Adelson, J. L., & Hautzinger, M. (2011). A randomized trial to evaluate the course of effects of a program to prevent adolescent depressive symptoms over 12 months. *Behaviour Research and Therapy*, *49*(12), 838–851. https://doi.org/10.1016/j.brat.2011.09.010

Raimundo, R., Marques-Pinto, A., & Lima, M. L. (2013). THE EFFECTS OF A SOCIAL-EMOTIONAL LEARNING PROGRAM ON ELEMENTARY SCHOOL CHILDREN: THE ROLE OF PUPILS’ CHARACTERISTICS: Effects of a Social-Emotional Learning Program. *Psychology in the Schools*, *50*(2), 165–180. https://doi.org/10.1002/pits.21667

Spilt, J. L., Koot, J. M., & Van Lier, P. A. C. (2013). For Whom Does It Work? Subgroup Differences in the Effects of a School-Based Universal Prevention Program. *Prevention Science*, *14*(5), 479–488. https://doi.org/10.1007/s11121-012-0329-7

Sullivan, T. N., Sutherland, K. S., Farrell, A. D., Taylor, K. A., & Doyle, S. T. (2017). Evaluation of Violence Prevention Approaches Among Early Adolescents: Moderating Effects of Disability Status and Gender. *Journal of Child and Family Studies*, *26*(4), 1151–1163. https://doi.org/10.1007/s10826-016-0629-9

Vroom, E. B., Massey, O. T., Yampolskaya, S., & Levin, B. L. (2020). The Impact of Implementation Fidelity on Student Outcomes in the Life Skills Training Program. *School Mental Health*, *12*(1), 113–123. https://doi.org/10.1007/s12310-019-09333-1

Wigelsworth, M., Humphrey, N., & Lendrum, A. (2013). Evaluation of a School-wide Preventive Intervention for Adolescents: The Secondary Social and Emotional Aspects of Learning (SEAL) Programme. *School Mental Health*, *5*(2), 96–109. https://doi.org/10.1007/s12310-012-9085-x

Wigelsworth, M., Squires, G., Birchinall, E., Kalambouka, A., Lendrum, A., Black, L., Troncoso, P., Santos, J., Ashworth, E., & Britteon, P. (2018). *FRIENDS for life: Evaluation report and executive summary.* Education Endowment Foundation.

Witvliet, M., Van Lier, P. A. C., Cuijpers, P., & Koot, H. M. (2009). Testing links between childhood positive peer relations and externalizing outcomes through a randomized controlled intervention study. *Journal of Consulting and Clinical Psychology*, *77*(5), 905–915. https://doi.org/10.1037/a0014597
